# Single-cell transcriptional mapping of *Mustn1* exhibits consistent mural cell localization across musculoskeletal tissues

**DOI:** 10.1093/jbmrpl/ziaf193

**Published:** 2025-12-18

**Authors:** Christopher J Janton, Anne Nichols, Michael Hadjiargyrou

**Affiliations:** Department of Biological and Chemical Sciences, New York Institute of Technology, Old Westbury, NY 11568, United States; College of Osteopathic Medicine, New York Institute of Technology, Old Westbury, NY 11568, United States; Department of Orthopedics, University of Rochester, Rochester, NY 14627, United States; Department of Biological and Chemical Sciences, New York Institute of Technology, Old Westbury, NY 11568, United States

**Keywords:** musculoskeletal temporally activated novel gene (*Mustn1*), alpha smooth muscle actin (*aSMA*), single-cell RNA sequencing (scRNA-seq), perivascular cell, mural cell, vascular smooth muscle cell (vSMC), fibroadipogenic progenitors (FAPs), skeletal muscle, bone, tendon

## Abstract

The musculoskeletal temporally activated novel gene (*Mustn1*) is a 9.2-kDa microprotein that has been extensively studied within musculoskeletal tissues. Utilizing open-source, single-cell RNA sequencing datasets, we used a top-down, transcriptional approach that combines systems-wide and targeted-tissue mapping of *Mustn1*. In doing so, we observed robust mural cell-state colocalization of Mustn1 and Acta2 (encoding alpha smooth muscle actin [aSMA]) within bone, synovium, muscle, and tendon tissues. Mapping *Mustn1* within recently documented, uncharacterized cell clusters of Sox9 lineage also revealed overlap with musculoskeletal fibroadipogenic progenitors (FAPs) and mural cells. Overall, these findings demonstrate that aSMA-expressing cells of the periosteum and tendon sheath provide a highly selective cell population to leverage the study of Mustn1 during musculoskeletal development and repair.

## Introduction

The emergence of single-cell (sc) transcriptomics^1^ (scRNA sequencing) has given researchers unprecedented ability to characterize cellular heterogeneity. The previous mainstay of transcriptional analyses—that is, bulk RNA sequencing (RNA-seq)—allowed for deeper sequencing reads of a targeted sample, but lacked the ability to distinguish the cellular populations responsible for contributing to underlying transcriptional read-outs. Single-cell methodologies, although limited in their depth of sequencing abilities, provide alternative strengths in discerning the contribution of cell types within a sequenced sample.^2^ As open-source data deposition within the scientific community has enabled these datasets to be repurposed well beyond their initial intentions, it is imperative to cross-reference prior reports with newly available data atlases. This retrospective connection can be used to generate higher resolution hypotheses that are seeded in previous reports.

In this study, we analyzed open-source, sc transcriptomics datasets through the lens of *Mustn1* to localize which cell populations underpin expression throughout the hindlimb. *Mustn1* was originally identified and cloned during studies of rat bone regeneration within our laboratory,^3^^,^^5^ and its expression has been reported exclusively in vertebrate animals by us as well as others. Specifically, *Mustn1* is located on chromosome 14 in mice, codes for an 82 amino acid protein (9.2 kDa).^3–6^ Although polypeptide sequence^4^ and structure predictions^6^ across vertebrate species show a range of homology, *Mustn1* expression has been extensively documented in the skeletal muscle of multiple species, including the following: zebrafish,^7^^,^^8^ trout,^9^ ducks ^10^^,^^11^, chickens,^12–15^ pigs,^16–18^ dogs,^19^ minks^20^, rodents^21–27^, and humans.^28^ Recent reports show that Mustn1 is a microprotein secreted from the smooth muscle cells (SMCs) of skeletal muscle, highlighting an effect in extracellular matrix remodeling within *Mustn1*-deficent muscle during hindlimb reloading.^22^  *Mustn1* has also been identified in other musculoskeletal tissues, such as cartilage^29^^,^^30^ and tendon/ligaments of humans^31^^,^^32^ and rodents.^4^^,^^33^ Although scarce, reports of *Mustn1* within other tissues are beginning to emerge. Specifically, recent reports have documented its expression in the mouse aorta,^22^ pig fat tissue,^34^ human testicular tissue,^35^ and infantile hemangioma^36^ and genome-wide association study assays have highlighted the *Mustn1* locus within human neural tissue.^37^ As *Mustn1* expression has emerged within the vasculature of various tissues, its localization within blood vessels of other musculoskeletal tissues—that is, tendon and bone—remains to be determined.

Herein, we leverage open-source scRNA sequencing datasets to demonstrate that *Mustn1* expression highly correlates with alpha smooth muscle actin (*aSMA*; *Acta2*) within mural cell populations throughout the mouse hindlimb. We hope this top-down and targeted-tissue mapping approach may provide a framework for investigating scientific genes-of-interest using widely available graphical user interfaces (GUIs) and uploaded Gene Expression Omnibus (GEO) data matrices. Moreover, these findings demonstrate the uniformity of perivascular *Mustn1* expression across hindlimb tissue types. This highlights the need to investigate how perivascular *Mustn1* expression within bone and tendon may affect extracellular matrix remodeling and fibrotic remodeling.

## Materials and methods

### Mice

*Sox9*-CreER,^38^ Ai9-tdTomato^39^ transgenic mice were obtained from Dr. Henry M. Kronenberg in the Endocrine Unit at Massachusetts General Hospital.^40^ Mice were previously back-crossed into C57/Bl6 mice for at least 5 generations. All animal care and experiments were carried out in accordance with the guidelines of the Institutional Animal Care and Use Committee (New York Institute of Technology)–approved protocols and met or exceeded all federal guidelines for the humane use of animals in research. The animal room was maintained at 21°C, 50% humidity, and a 12-hour per 12-hour light-dark cycle. All mice had access to standard chow and water ad libitum, as well as enrichment material (eg, nesting squares and plastic housing).

### Tamoxifen injection

Transgenic mice were pulsed with 75 mg/kg tamoxifen (MedChemExpress, ICI 47699) via a singular, bolus intraperitoneal injection at postnatal (p)21 and euthanized at p49 (4-wk chase). Tamoxifen was initially dissolved in 100% ethanol; sunflower oil (Sigma Aldrich, S5007) was then added, and the ethanol was evaporated out overnight at 60°C.

### Histology

Mice were euthanized via CO_2_ asphyxiation. For neuronal tissue, brains were harvested from p28 C57Bl/6 mice and placed into 4% paraformaldehyde (PFA) overnight at 4°C. Hindlimbs were separated at the femoral head and placed into 4% PFA (Sigma, 158 127) overnight at 4°C. Sox9-lineage mice were euthanized as described above. For co-immunostaining Mustn1 and aSMA, described below, p28 C57Bl/6 mice were used. Tissue was immediately transferred to 30% sucrose for 24 hours and embedded into Optimal Cutting Temperature (OCT) compound (Fisher 4585). For neuronal tissues, 35-μm cryosections were cut on a Leica CM 1950 and placed free-floating into PBS. Hindlimb cryosections (12-μm thickness) were captured with cryotape method^41^ using a Leica CM 1950.

### Immunofluorescence

Brain and hindlimb tissue slides were washed with PBS then blocked with 5% BSA (Sigma A9418), 0.05% Tween 20 (Sigma P2287), 1 mM CaCl_2_ in PBS for 1 hour at room temperature. Primary rabbit anti-Mustn1 (1:500; Millipore ABD115) was incubated overnight at 4°C. Subsequently, mouse anti-SMA-Cy3 (Sigma C6198) and donkey anti-rabbit-IgG-647 (ThermoFisher catalog no. A-31573) were incubated for 1 hour at room temperature. Slides were washed with PBS and mounted with DAPI (Southern Biotech, 0100-20). Fluorescent images were acquired with Zeiss LSM 980. Image processing was performed using ZEN Lite. Far-red (Mustn1) was pseudo-colored green to improve clarity.

### Systems-wide transcriptional mapping

Large-scale, sc sequencing atlases, such as The Human Protein Atlas,^42^^,^^43^ the CZ CellxGene Discover,^44^ and Tabula Muris,^45^ were used to obtain a systems-wide readout of *Mustn1* expression in both human and mouse tissues.

### Targeted transcriptional mapping

Two approaches were used to access published, GEO-deposited sc transcriptomic data. The first utilized GUIs of tissue-specific cell atlases for human and mouse neuronal tissue,^42–49^ mouse muscle tissue,^50^ mouse tendon tissue,^33^ mouse bone tissue^51^ and mouse synovial tissue.^52^ If a GUI was not provided, GEO count matrices (mouse long bone,^53^ GSE156636 and Prx1-lineage muscle,^54^ GSE164573) and annotated Robjects (mouse muscle;^55^  https://datadryad.org/dataset/10.5061/dryad.t4b8gtj34) were examined and analyzed via Rstudio using the Seurat package.^56^ Annotated Seurat reductions for mouse muscle object^55^ were viewed as reported in the literature. Data on mouse long bone^53^ were filtered to exclude genes that were not present across a minimum of 3 cells and to exclude cells that do not have a minimum of 500 features, with a maximum of 4000 features and a total gene count of less than 20 000. Data on mouse Prx1-lineage muscle^54^ were filtered to exclude genes that were not present across a minimum of 3 cells and to exclude cells that do not have a minimum of 350 features, with a maximum of 8000 features and a total gene count less than 20 000. No mitochondrial genes were detected in uploaded matrices. Data were then log-normalized. Clustering was performed with scaled data on 2000 highly variable genes. Cells were clustered using principal components analysis (PCA) dimensionality of 10, as determined by elbow plot approximation, in combination with Louvian algorithms. Clustering was visualized via Uniform Manifold Approximation Projections (UMAP) and gene expression was visualized using dot and violin plots. The following gene markers were used to establish clustering annotations for *Prx1*-lineage cells as detailed in Julien et al.^54^: pericytes (*Mylk*, *Des*, *Cspg4*), tenocytes (*Kera*, *Scx*, *Tnmd*), and fibroadipogenic progenitors (FAPs) (*Prrx1, Cxcl12, Pdgfra, Ly6a, Cd34*). For visualization of Sox9-lineage cells within the mouse hindlimb,^40^ published clustering annotations were analyzed and expression plots were generated using Python via the Pegasus package.^57^ All R/python code is detailed stepwise in the Supplementary methods. For visualization of human peritendinous scar data, plots were generated by Dr. Nichols, as described in Nichols et al.^58^

## Results

### Mural cell expression of *Mustn1* in neuronal and synovial tissues

**Figure 1:**
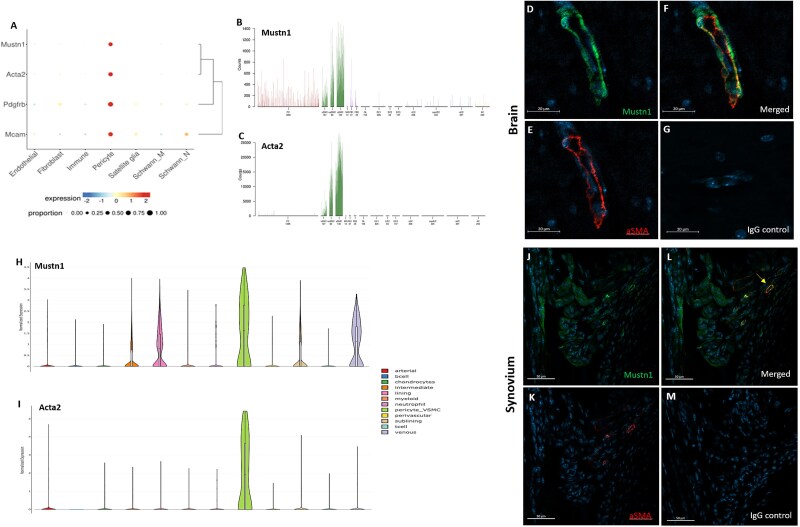
*Mustn1* and *aSMA* colocalization in neuronal and synovial tissue. (A) Dot plot showing *Mustn1*, *Acta2*, *Pdgfrb*, and *Mcam* coexpression in nonneuronal cells from dorsal root ganglia of humans, mice, and rodents, modified from Bhuiyan et al.^46^ (B) Bar plot showing expression of *Mustn1* and (C) *Acta2* expression across cell types of mouse brain, modified from Vanlandewijck et al^48^ and He et al.^49^ (D) Mustn1 (green) and (E) aSMA (red) immunostaining of p28 mouse neuronal vasculature. (F) Merged images showing aSMA (red) and Mustn1 (yellow) colocalization. (G) IgG isotype control. (H) Violin plots of Mustn1 and (I) Acta2 expression among cell types from inflamed mouse synovium, modified from Wei et al.^52^ (J) Mustn1 and (K) aSMA immunostaining. (L) Merged images demonstrating colocalization (yellow arrow) in the retrocalcaneal bursa of p28 mice. (M) IgG isotype control. Representative immunofluorescent images of *n* = 3 mice, DAPI stain (blue). Scale bar in panels D–G = 20 μm and in J–M = 50 μm. Abbreviations: aSMA, alpha smooth muscle actin; IgG, immunoglobulin G.

When gene expression was mapped within cells from the dorsal root ganglia of subtyped human and mouse tissue, modified from Bhuiyan et al,^46^  *Mustn1* exhibited coexpression with *Acta2*, *Pdgfrb*, and *Mcam* in the pericyte cell cluster (Figure 1A). In a separate neuronal atlas, constructed by Vanlandewijck et al.^48^ and He et al.^49^ examining neuronal cell types that have been enriched for vasculature and vessel-associated cell types, *Mustn1* (Figure 1B) and *Acta2* (Figure 1C) were highly coexpressed in arterial and venous SMCs. Among this vascular-enriched, neuronal dataset, *Mustn1*, but not *Acta2*, was expressed at a lower level among pericytes . Systems-wide mapping of *Mustn1* uniformly highlighted brain perivascular cells across data atlases (Figure S1). Co-immunostaining of Mustn1 (Figure 1D) and aSMA (Figure 1E) demonstrates positive co-localization in the vasculature of the brain cortex (Figure 1F). No signal was detected with the negative immunoglobulin G (IgG) isotype control (Figure 1G).

Gene mapping among synovial cell clusters isolated from inflamed arthritic tissue, modified from Wei et al,^52^ revealed that Mustn1 (Figure 1H) and Acta2 (Figure 1I) have the highest expression within perivascular cells. Mustn1 expression was also noted within synovial lining layer and venous cell populations. Immunostaining of Mustn1 (Figure 1J) and aSMA (Figure 1K) reveal co-expression within synovial vasculature, indicated by the yellow arrow (Figure 1L). No signal was detected with the negative IgG isotype control (Figure 1M).

### Robust co-expression of *Acta2* and *Mustn1* in femoral periosteum

**Figure 2:**
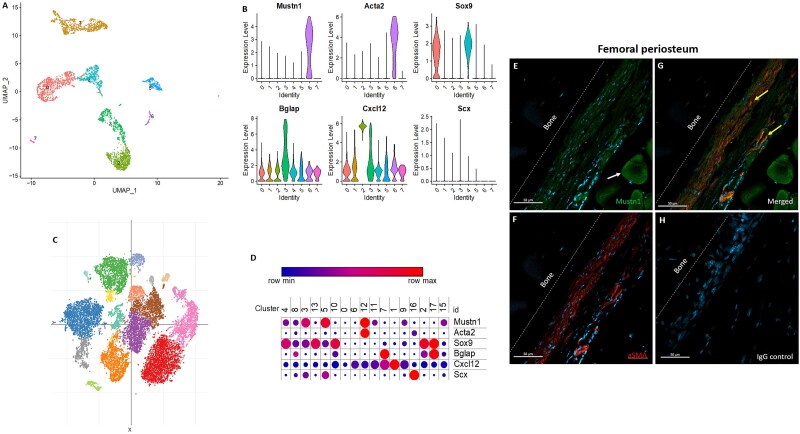
*Mustn1* and *aSMA* colocalization in femoral periosteum. (A) UMAP of 7 cellular clusters and (B) mapped violin plot expression of *Mustn1*, *Acta2*, *Sox9*, *Bglap*, *Cxcl12*, and *Scx* from p21 mouse long bones of the hindlimb, modified from Sivaraj et al.^53^ (C) t-distributed stochastic neighbor embedding (tSNE) projection of 17 cellular clusters and (D) dot plot showing expression of *Mustn1*, *Acta2*, *Sox9*, *Bglap*, *Cxcl12*, and *Scx* among hematopoietically inclusive mouse long bones, modified from Baryawno et al.^51^ (E) Immunostaining of Mustn1 (green) and (F) aSMA (red) of p28 femoral periosteum and adjacent skeletal muscle. The white arrow in panel E indicates Mustn1 expression in skeletal muscle. (G) Merged fluorescent images display regions of co-expression (yellow arrows). (H) IgG isotype control. Representative immunofluorescent images of *n* = 3 mice, DAPI stain (blue). Scale bar = 50 μm. Abbreviations: aSMA, alpha smooth muscle actin; DAPI, 4',6-diamidino-2-phenylindole; IgG, immunoglobulin G; max, maximum; min, minimum; UMAP, Uniform Manifold Approximation Projection.

Sequencing reads, generated by Sivaraj et al.^53^ examining hematopoietic-depleted cells from the femur and tibia of a p21 mouse, were run through a Seurat pipeline and a UMAP plot was generated (Figure 2A). When gene expression was mapped onto these clusters, *Mustn1* colocalized exclusively to cluster 6, alongside expression of *Acta2* (Figure 2B).

When we mapped expression on a separate skeletal dataset that included bone marrow stromal cells, modified from Baryawno et al.^51^ (Figure 2C), *Mustn1* expression was found to be highly correlated with *Acta2* and in cluster 12, defined by the authors to be pericytes (Figure 2D). Mustn1 was also expressed in fibroblast clusters 3 and 5 (tendon/ligament cells), with light expression in cluster 4 (chondrocyte progenitors), cluster 8 (MSC osteolineage cells), and cluster 11 (arterial endothelial cells). Immunostaining of Mustn1 (Figure 2E*)* and aSMA (Figure 2F*)* within the hindlimb of p28 mice revealed periosteal co-expression, indicated by yellow arrows (Figure 2G*)*. Mustn1 signal was also detected in the adjacent muscle fibers (Figure 2E, white arrow). No signal was detected with the negative IgG isotype control (Figure 2H).

### *Mustn1* is expressed within both vascular cells and muscle fiber cells of the tibialis anterior

**Figure 3:**
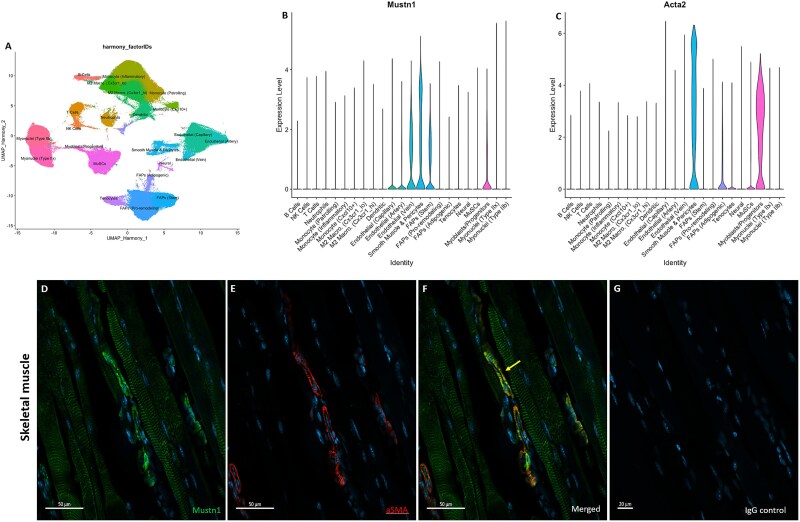
*Mustn1* and *aSMA* colocalization in skeletal muscle. (A) UMAP plot of sc muscle cell annotations with violin plots showing mapped (B) *Mustn1* and (C) *Acta2* transcriptional expression, modified from McKellar et al.^55^ p28 Tibialis anterior immunostaining of (D) Mustn1 (green), (E) aSMA (red), and (F) merged images that indicate co-expression (yellow arrow). (G) IgG isotype control. Representative immunofluorescent images of 3 mice, DAPI stain (blue). Scale bar in panels D–F = 50 μm and in G = 20 μm. Abbreviations: aSMA, alpha smooth muscle actin; DAPI, 4',6-diamidino-2-phenylindole; FAP, fibroadipogenic progenitor; IgG, immunoglobulin G; max, maximum; min, minimum; UMAP, Uniform Manifold Approximation Projection.

Gene expression was explored in a comprehensive mouse muscle atlas, modified from McKellar et al*,*^55^ consisting of integrated single-nucleus and sc RNA-seq datasets from various experiments (Figure 3A). As shown recently,^22^  *Mustn1* expression was detected in SMCs and pericytes, with notable, low expression in endothelial cells (capillary, artery, and vein) and FAPs (FAPs Stem) (Figure 3B). *Acta2* expression was also highlighted within SMCs and pericytes (Figure 3C*)**.***

*Mustn1* was mapped onto constituent datasets^49^ of the comprehensive mouse atlas^55^ to test for robustness in the observed expression profile of *Mustn1*. *Mustn1* expression was observed in p21 mouse tibialis anterior (TA) muscle (Figure S2A), within myotendinous junction (MTJ) and myonuclei clusters but not within SMCs or pericytes (Figure S2B). When muscle cells from a 30-mo-old TA (Figure S2C*)* were analyzed, *Mustn1* was observed solely in the myonuclei clusters of the mouse TA (Figure S2D). Surprisingly, *Mustn1* was not reported in any cell clusters within the p10, 5-mo, or 24-mo TA mouse muscle, or the 5-mo soleus mouse muscle datasets (data not shown).

Gene expression was then mapped onto a mesenchymal-enriched, muscle dataset^59^ (Figure S2E), not included as a constituent of the comprehensive muscle atlas. Low *Mustn1* expression was found in all mesenchymal clusters of the muscle, with the highest expression being within the pericyte and vascular SMC cluster (Figure S2F*)*.

To demonstrate the expression of Mustn1, we used the tibialis anterior muscle of a p28 C57Bl/6 mouse. Immunostaining for Mustn1 (Figure 3D*)* and aSMA (Figure 3E*)* show both proteins colocalized within muscular vasculature as indicated by the yellow arrow (Figure 3F*)*. Mustn1 signal can also be seen within adjacent muscle fibers (Figure 3D and F*)*. No signal was detected with the negative IgG isotype control (Figure 3G).

### Expression of *Mustn1* by tenocytes and mural cells

**Figure 4:**
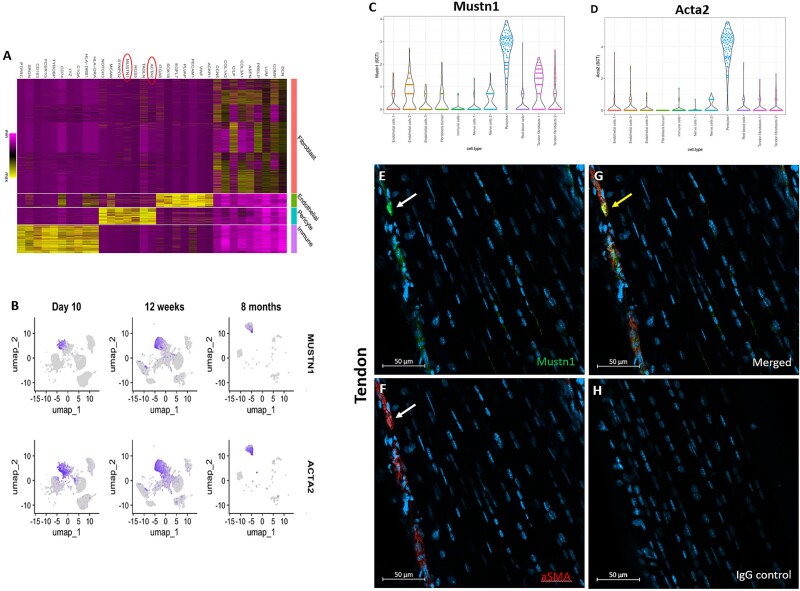
*Mustn1* and *aSMA* colocalization in tendon and ligaments. (A) Heat map, modified from Yang et al,^41^ showing *Mustn1*, *Acta2* (red circles) expression within pericyte cell state of human anterior cruciate ligaments. (B) UMAP expression projections of *Mustn1* and *Acta2* transcripts within 10 d, 12 wk, and 8 mo postinjury, peritendinous human scar tissue (Nichols et al.^58^). (C) Violin plot showing *Mustn1* and (D) *Acta2* expression within cell clusters of a 6-wk mouse Achilles tendon, modified from De Micheli et al.^33^ Immunofluorescent staining of (E) Mustn1 (green; white arrow) and (F) aSMA (red; white arrow) within p28 Achilles tendon. (G) Merged images showing colocalization (yellow arrow). (H) IgG isotype control. Representative immunofluorescent images of 3 mice, DAPI stain (blue). Scale bar = 50 μm. Abbreviations: aSMA, alpha smooth muscle actin; DAPI, 4',6-diamidino-2-phenylindole; IgG, immunoglobulin G; max, maximum; min, minimum; UMAP, Uniform Manifold Approximation Projection.

In the human anterior cruciate ligament, *Mustn1* was reported as a differentially expressed marker gene within the pericytes^41^ (Figure 4A**)**. When mapped onto human peritendinous scar tissue at 10 d, 12 wk, and 8 mo postinjury,^58^  *Mustn1* exhibited high co-expression alongside *Acta2*-expressing cell clusters (Figure 4B). Within the Achilles tendon of a 6-wk-old mouse,^33^  *Mustn1* expression was highest in pericytes with slight expression in endothelial cell 2 and tendon fibroblast 1 (tenocyte) clusters (Figure 4C), while *Acta2* localized exclusively to the pericyte cluster (Figure 4D). Mustn1 immunostaining highlights low expression by some tenocytes (Figure 4E, white arrow). Immunostaining for aSMA localized exclusively to the paratenon of the Achilles tendon of a p28 C57Bl/6 mouse (Figure 4F). Last, Mustn1 signal colocalized with aSMA in the paratenon, as indicated by the yellow arrow (Figure 4G*,* yellow arrow*)*. No signal was detected with the negative IgG isotype control (Figure 4G).

### *Mustn1* expression within tenocyte and mural cell clusters of hindlimb mesenchymal lineages

**Figure 5:**
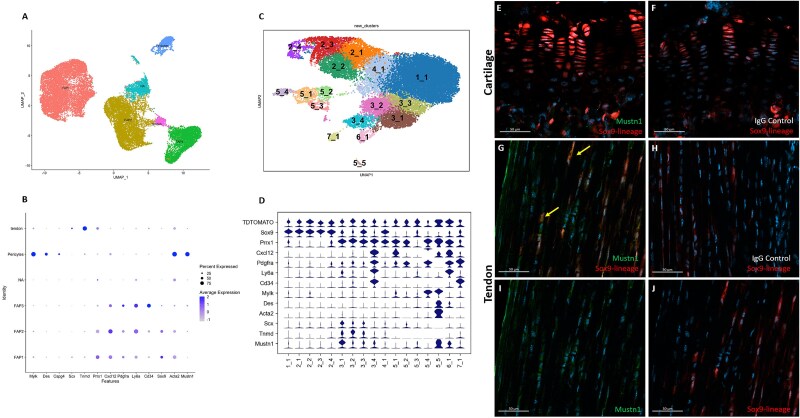
*Mustn1* within the mesenchymal lineages of the mouse hindlimb. (A) UMAP plot and (B) expression dot plot from *Prx1*-lineage cells isolated from skeletally proximal muscle in the setting of noninjury, postfracture, and post-polytrauma, modified from Julien et al.^54^ (C) UMAP plot and (D) violin plot showing TdTOMATO, *Sox9*, *Prx1*, *Cxcl12*, *Pdgfra*, *Ly6a*, *Cd34*, *Mylk*, D*es*, *Acta2*, *Scx*, *Tnmd*, and *Mustn1* expression in cell clusters descendent from Sox9-lineage cells of the mouse hindlimb, modified from Smith et al.^40^ Epiphyseal plate immunofluorescent images of (E) Mustn1 (green) staining alongside p21-p49 descendants of Sox9-lineage cells (red) and (F) IgG isotype control. Achilles tendon immunofluorescent images of (G) merged images highlighting co-expression (yellow arrows) of (I) Mustn1 (green) staining alongside (J) Sox9-lineage (red) tenocytes. (H) IgG isotype control. Representative immunofluorescent images of 3 mice, DAPI stain (blue). Scale bar = 50 μm. Abbreviations: DAPI, 4',6-diamidino-2-phenylindole; FAP, fibroadipogenic progenitor; IgG, immunoglobulin G; UMAP, Uniform Manifold Approximation Projection.

Sequencing reads, constructed by Julien et al.^54^ from *Prx1*-lineage cells that were isolated from TA muscle adjacent to uninjured and fractured skeletal tissue, were run through a Seurat pipeline and clustered (Figure 5A). Using the published genetic markers, *Mustn1* expression was robustly observed in pericyte cell clusters along with *Mylk*, *Des*, and *Cspg4* (Figure 5B). Very minimal signal was detected in tendon and FAP clusters. Within the recently published *Sox9*-lineage cells of the mouse hindlimb^40^ (Figure 5C), *Mustn1* was co-expressed with *Scx* and *Tnmd* in defined tenocyte clusters 3_1, 3_2 and 3_3, as well as *Acta2*, *Mylk*, and *Des* in undefined cluster 5_5. Minimal Mustn1 expression was observed within undefined clusters 3_4 and 6_1 expressing *Cxcl12*, *Pdgfra*, *Ly6a*, and *Cd34* (Figure 5D). When immunostaining the hindlimb, Mustn1 was not observed within Sox9-lineage chondrocytes of the epiphyseal plate (Figure 5E). Colocalization (yellow arrow, Figure 5G) of Mustn1 immunostaining (Figure 5I) with *Sox9*-lineage (Figure 5J) was observed within tenocytes of the p28 mouse Achilles tendon. The negative IgG isotype control showed only the red color from the *Sox9*-lineage (Figure 5F and H).

## Discussion

Although systems-wide mapping of *Mustn1* demonstrated high mural cell expression in the brain, these large databases contain a skewed number of soft tissue cell types vs cells of the hindlimb—that is, 3.5 million mouse brain cells vs 50 thousand hindlimb cells. Prior literature on *Mustn1* focused predominantly on the tissues of the hindlimb, especially skeletal muscle. In this study, we detail a top-down, systems-wide mapping approach of *Mustn1*, accompanied by targeted-tissue analyses of the hindlimb. Our findings indicate that *Mustn1* expression is strongly correlated with the cellular expression of aSMA within mural cells of the synovium, muscle, bone, and tendon as well as brain.

Most recently, *Mustn1* expression within the muscle was shown to have high perivascular expression,^22^ as well as light expression in FAPs, myonuclei, and progenitor myoblasts. We compared this *Mustn1* expression profile with subsequent, constituent datasets to test for the robustness of transcriptional readout. To our surprise, there was no smooth muscle expression within isolated, single-nucleus transcriptomes of p21 and p30 TA cells. This SMC expression was, however, observed within a nonconstituent muscle dataset. The cumulative atlas contains transcripts representative of 365 000 nuclear and cellular transcriptomes, while the p21 TA dataset contains 11 552 nuclear transcriptomes and the mesenchymal-enriched muscle dataset was constructed with only 1754 cellular transcriptomes. Although smaller in sample size, and not included in the cumulative atlas, the perivascular expression within mesenchymal-enriched muscle cells matches with Mustn1 expression within the large, cumulative atlas, as is confirmed by colocalization of *Mustn1* and *aSMA* proteins within the muscular vasculature. The lack of *Mustn1* within single-nuclear transcriptomes may point to differences in the amount of nuclear and cytoplasmic *Mustn1* transcripts.

The muscular mural cell expression of *Mustn1* is also observed within perivascular cells of the *Prx1*-lineage in muscle tissue adjacent to the femur. During fracture or polytrauma (fracture, plus muscle damage), this cluster of perivascular cells remained unchanged in number and expression profile.^54^ In contrast, the FAPs from the muscle, particularly those of the Prg4-lineage,^60^ were shown to play a critical role in osteochondral differentiation after musculoskeletal perturbation. When mapping cluster-specific gene markers from the *Prx1*-lineage onto the *Sox9*-lineage, we noted high overlap between *Prx1*-lineage FAP clusters and uncharacterized clusters 3_4, 6_1 and 7_1 of the *Sox9*-lineage, indicating that these novel clusters may characterize as FAPs.

*Mustn1* was originally cloned as a novel upregulated gene during skeletal fracture repair.^6^ Transcriptional localization to the periosteum of intact mouse long bone and periosteal cells postfracture was previously reported by our laboratory.^3^ Within our mappings of the mouse long bone, *Mustn1* demonstrates high localization to Acta2-positive cell populations, This *Mustn1–Acta2* correlation is also seen in the *Sox9*-lineage of the mouse hindlimb, particularly in the uncharacterized cells of cluster 5_5. As such, there are 2 possibilities that may explain these results. One is that cluster 5_5 of the *Sox9*-lineage retains *Acta2*-positive periosteal cells, and the other is that cluster 5_5 may harbor perivascular cells within the periosteum, as highlighted in a recent characterization of periosteal heterogeneity.^61^ When analyzing the expression of aSMA and Mustn1, we observed strong colocalization within femoral periosteal cells. Previously, it was shown that the *Sox9*-lineage and *Acta2*-lineage of skeletal cells labels cell populations within the periosteum of uninjured long bones and that, during fracture repair, both cell lineages expand greatest during early skeletal regeneration.^61^ The upregulation of *Mustn1* first observed in early skeletal repair may be due to an increase in *Sox9-/Acta2*-lineage periosteal cells, although this hypothesis requires further testing.

When analyzing cellular expression from cells of a long bone with intact hematopoietic fraction, *Mustn1* overlapped with Sox9-expressing tendon/ligament cells, but not in Sox9-expressing chondrocytes. We did not observe *Mustn1* expression in chondrocyte clusters 1_1 to 2_4 of the Sox9-lineages*,* nor did we observe immunostaining within the epiphyseal plate. However, we did observe *Mustn1* transcripts within tenocyte clusters of the *Sox9*-lineage and Mustn1 protein colocalized by immunofluorescence to *Sox9*-lineage tenocytes on the Achilles tendon. By cross-referencing these different skeletal datasets, we confirmed the robustness of cell profiles across these skeletal transcriptomes.

*Mustn1* expression in the tendon has been previously reported^3^ but, to our knowledge, never explored. Herein, we show that mural cells and tenocytes underpin the expression of *Mustn1*, with the highest *Mustn1* expression being within aSMA-positive paratenon cells. Recent fate-mapping experiments using *Pdgfrb*-lineage cells within the healing mouse Achilles tendon showed infiltration during early tendon healing but were distinct from *Acta2*-positive myofibroblasts by 14 d after injury.^62^ The *Axin2*-lineage of cells within the Achilles tendon shows a much higher overlap with Acta2-positive myofibroblasts at 10, 20, and 30 d postinjury.^63^ It is unknown whether *Mustn1* is expressed in responsive myofibroblasts of the tendon, but *Acta2*-lineage cells would provide a targeted cell population to answer this, a question we are actively exploring.^64^

In addition to the other tissues analyzed here, *Mustn1* was reported to be one of the top differentially expressed genes for pericytes within the mouse kidney,^65^ alongside *Myh11*, *Mcam*, and *Acta2* genes. During nephrotic fibrosis of the kidney, it was shown that pericytes differentiate into most of the *aSMA*-expressing myofibroblasts responsible for fibrosis.^66^ The dynamics of *Mustn1* in the differentiation of fibrotic, *Acta2*-positive myofibroblasts should be investigated in the renal system, the musculoskeletal system, and beyond. Overall, our findings robustly demonstrate that *Mustn1* expression is high within mural cells across the various tissues of the musculoskeletal system. These findings can be leveraged by the scientific community to spotlight targeted cell populations for the study of *Mustn1* and its role in extracellular matrix remodeling during tissue repair.

## Supplementary Material

Supp_Fig1_ziaf193

Supp_Fig2_ziaf193

Supplementary_Methods_ziaf193

## Data Availability

The publicly available datasets used in the generation of this manuscript are detailed in the Materials and methods section.
