# Supplementary figures and images for: Single-cell transcriptional mapping of Mustn1 exhibits consistent mural cell localization across musculoskeletal tissues
Source: JBMR Plus. 2025 Dec 18;10(2):ziaf193. doi: 10.1093/jbmrpl/ziaf193 (PMC12790277; doi:10.1093/jbmrpl/ziaf193)

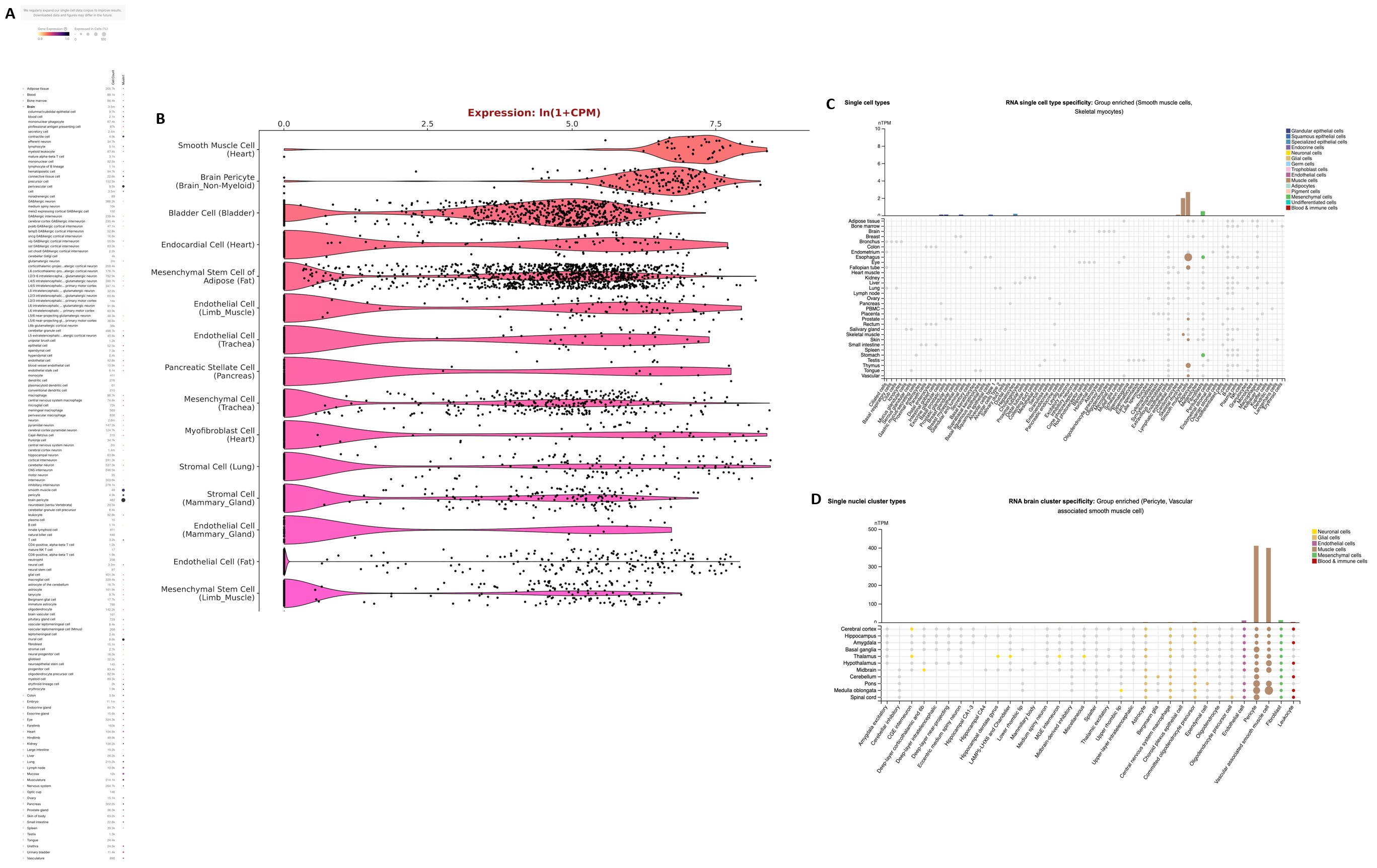

Supplement: Supp_Fig1_ziaf193 [file supp_fig1_ziaf193.jpeg]

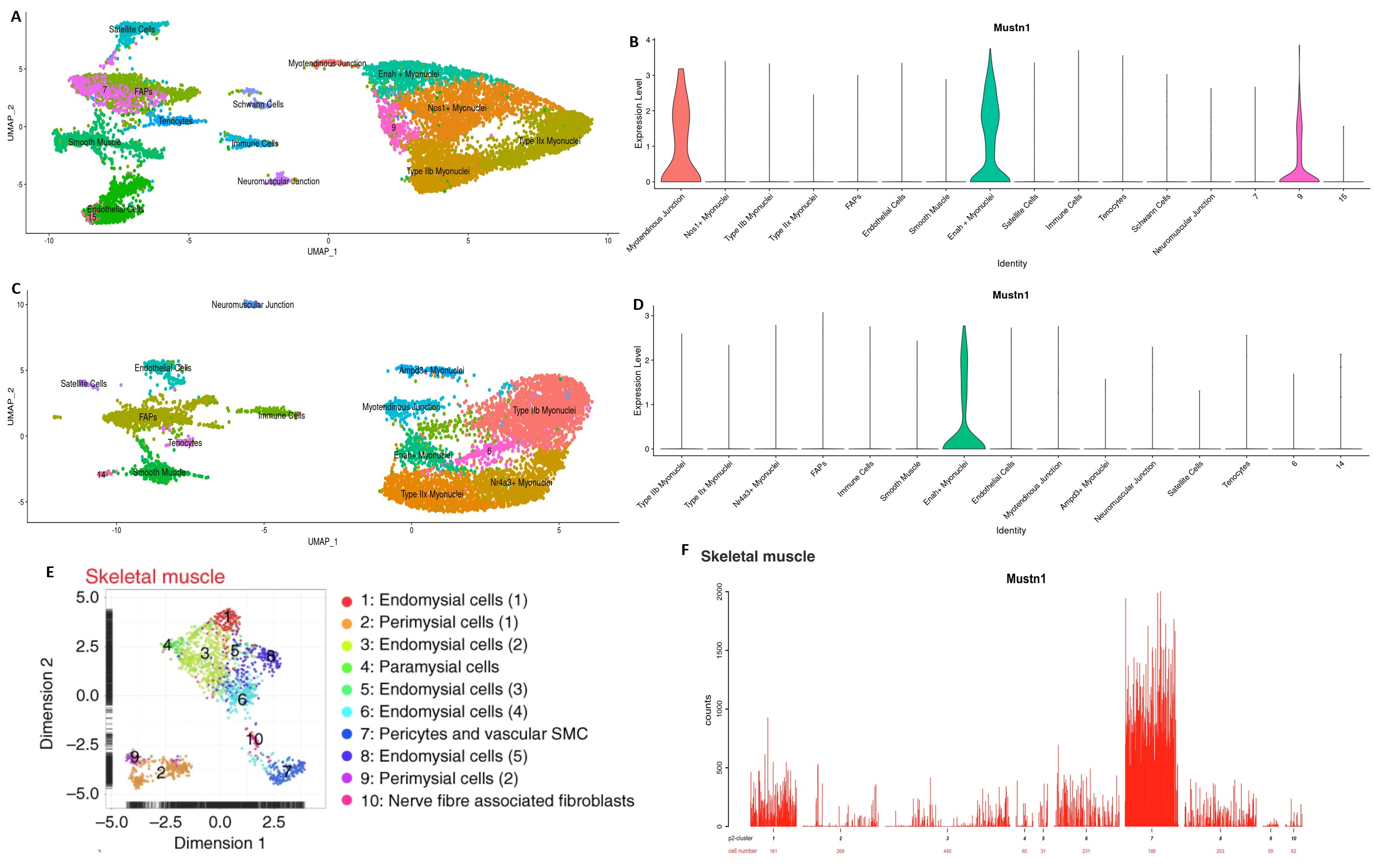

Supplement: Supp_Fig2_ziaf193 [file supp_fig2_ziaf193.jpeg]
