# Supplementary material for: Single-cell transcriptional mapping of Mustn1 exhibits consistent mural cell localization across musculoskeletal tissues
Source: JBMR Plus. 2025 Dec 18;10(2):ziaf193. doi: 10.1093/jbmrpl/ziaf193 (PMC12790277; doi:10.1093/jbmrpl/ziaf193)

**Supplementary Methods.**

*Mustn1 expression mapped onto the p21 mouse long bone^42^ provided by GSE156636.*

First, load the necessary Rstudio packages.

library(Seurat)
library(tidyverse)
library(Matrix)
library(data.table)

Data matrix was downloaded from GEO website (<https://www.ncbi.nlm.nih.gov/geo/query/acc.cgi?acc=GSE156636>) and then modified to create a sparse matrix compatible with Seurat

counts_df1 <- fread("/path/to/file/ GSM4735393_Bone-1_gene_counts.tsv.gz

", data.table = FALSE)
colnames(counts_df1) <- c("gene", "barcode", "count")
genes <- unique(counts_df1$gene)
barcodes <- unique(counts_df1$barcode)
gene_idx <- match(counts_df1$gene, genes)
barcode_idx <- match(counts_df1$barcode, barcodes)
counts_matrix1 <- sparseMatrix(
 i = gene_idx, # Row indices (genes)
 j = barcode_idx, # Column indices (barcodes)
 x = counts_df1$count, # Values (counts)
 dims = c(length(genes), length(barcodes)), # Dimensions of the matrix
 dimnames = list(genes, barcodes) # Row and column names
)
scTendonObject1 <- CreateSeuratObject(counts =
 counts_matrix1, project = "pdgfrbBone1", min.cells = 3, min.features = 200)
head(scTendonObject1@meta.data)

## orig.ident nCount_RNA nFeature_RNA
## AAGCAGTGGTATCAAC pdgfrbBone1 5543 5452
## AGATTGCCATGCAATC pdgfrbBone1 5448 2424
## CGCTTCAAGGGAGTAA pdgfrbBone1 12955 3329
## GCCTCTAAGTGTCCAT pdgfrbBone1 3893 1915
## GGAAAGCCATCGATTG pdgfrbBone1 4827 2127
## GTCACAACACGAGAGT pdgfrbBone1 1774 1115

Pre-QC data plotted for references.

#load mitochondrial DNA as Seurat object
scTendonObject1 <- PercentageFeatureSet(scTendonObject1, pattern = "^MT-", col.name = "percent.mt")
#plot pre-QC data
VlnPlot(scTendonObject1, features = c("nFeature_RNA", "nCount_RNA", "percent.mt"), ncol = 3)


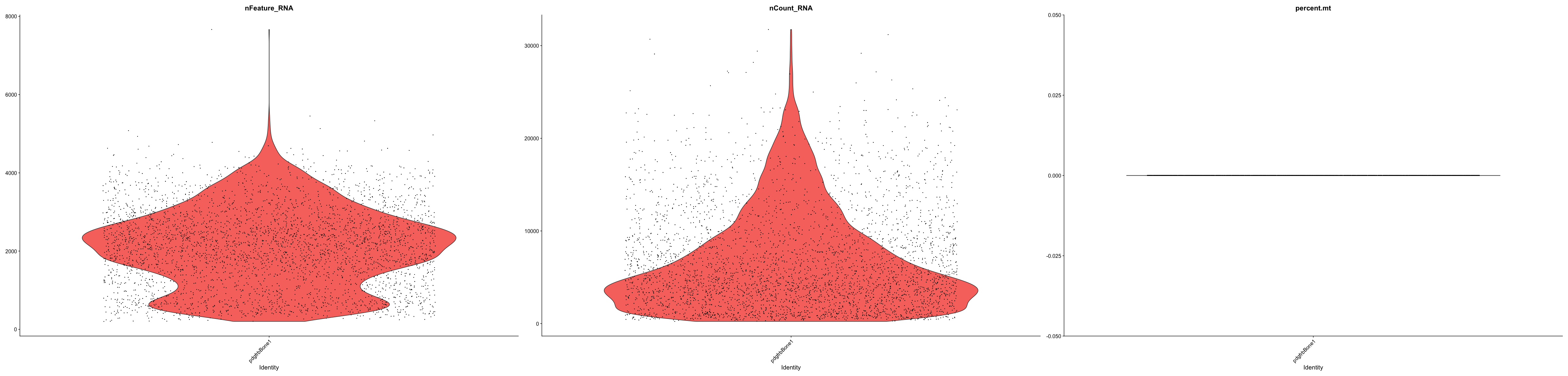


Next, quality control (QC) metrics were run as described in methods section and post-QC results are plotted for reference.

#QC filter data
scTendonObject1 <- subset(scTendonObject1, subset = nFeature_RNA > 500 &
 nFeature_RNA <4000 &
 nCount_RNA < 20000)
#plot post-QC data
VlnPlot(scTendonObject1, features = c("nFeature_RNA", "nCount_RNA", "percent.mt"), ncol = 3)


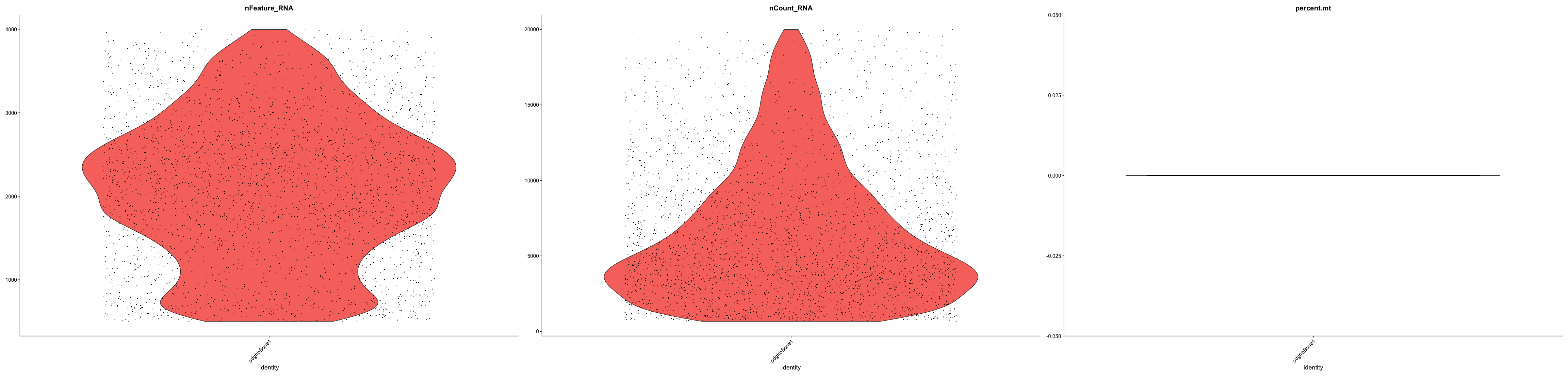


The data is then normalized logarithmically and the 2000 highest variable genes are identified.

#Data normalization
scTendonObject1 <- NormalizeData(scTendonObject1, normalization.method = "LogNormalize", scale.factor = 10000)
#identify highly variable features
scTendonObject1 <- FindVariableFeatures(scTendonObject1, selection.method = "vst", nfeatures = 2000)

Data scaling is applied and Principle Component Analyses (PCA) is performed. An elbow plot is used to determine the dimensionality of the data.

#data scaling
scTendonObject1 <- ScaleData(scTendonObject1)
all.genes <- rownames(scTendonObject1)
scTendonObject1 <- ScaleData(scTendonObject1, features = all.genes)

#Linear Dimensional reduction
scTendonObject1 <- RunPCA(scTendonObject1)
ElbowPlot(scTendonObject1, ndims = 50, reduction = "pca")


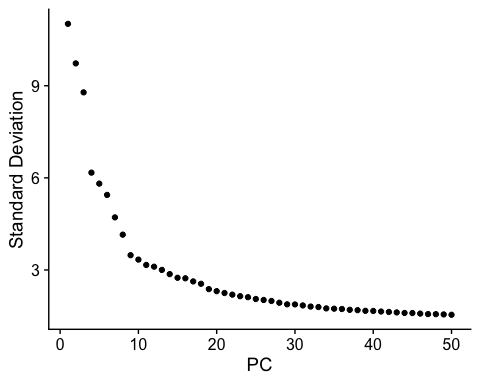


Cells are then clustered using PCA dimensionality of 10, as determined by hinge-point of elbow plot, in combination with Louvian algorithms.

#contruct K-nearest neighbor (KNN) graph using PCA dimensionality of 10
scTendonObject1 <- FindNeighbors(scTendonObject1, dims = 1:10)
#Cluster cells using Louvian algorithim with resolution of 0.5
scTendonObject1 <- FindClusters(scTendonObject1, resolution = 0.5)

## Modularity Optimizer version 1.3.0 by Ludo Waltman and Nees Jan van Eck
##
## Number of nodes: 3368
## Number of edges: 103729
##
## Running Louvain algorithm...
## Maximum modularity in 10 random starts: 0.9198
## Number of communities: 12
## Elapsed time: 0 seconds

Uniform Manifold Approximation and Projection (UMAP) was used to visualize clustering in low-dimensional space.

scTendonObject1 <- RunUMAP(scTendonObject1, dims = 1:10)
DimPlot(scTendonObject1, reduction = "umap", label = TRUE, repel = TRUE)

Mustn1, Sox9, Prg4, Mcam, Acta2, Pdgfrb and Myh11 coexpression plotted via violin plots.

VlnPlot(scTendonObject1, features = c("Mustn1", "Acta2", "Sox9",'Bglap','Cxcl12','Scx’)

**Mustn1 expression mapped onto the comprehensive mouse muscle atlas^38^, downloaded from: https://datadryad.org/dataset/doi:10.5061/dryad.t4b8gtj34 .**

When running on running a 16Gb RAM MacOS 13.6.7, it was necessary to run the following in terminal to open up RAM prior to loading annotated R object (11.97Gb) into local environment:

cd ~

touch .Renviron

open .Renviron

R_MAX_VSIZE = 100GB

Load necessary Rstudio packages and annotated data object.

library(parallel)
library(Seurat)
library(tidyverse)
scTendonObject1 <- load("/path/to/file/scMuscle_mm10_slim_v1-1.RData")

Plot UMAP cellular clusters and map Mustn1 onto published reductions.

DimPlot(scMuscle.slim.seurat, reduction = "umap_harmony", label = TRUE, repel = TRUE, group.by = "harmony_factorIDs", label.box = FALSE)


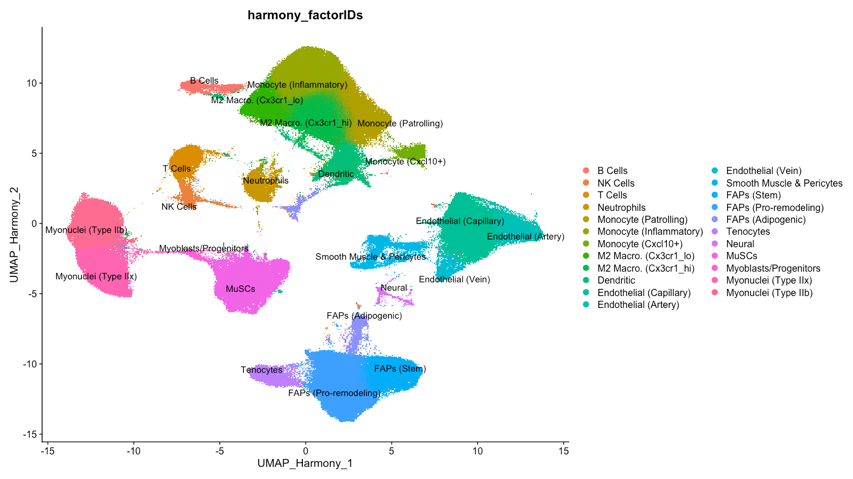


VlnPlot(scMuscle.slim.seurat, features = c("Mustn1"), group.by = "harmony_factorIDs")

VlnPlot(scMuscle.slim.seurat, features = c("Acta2"), group.by = "harmony_factorIDs")


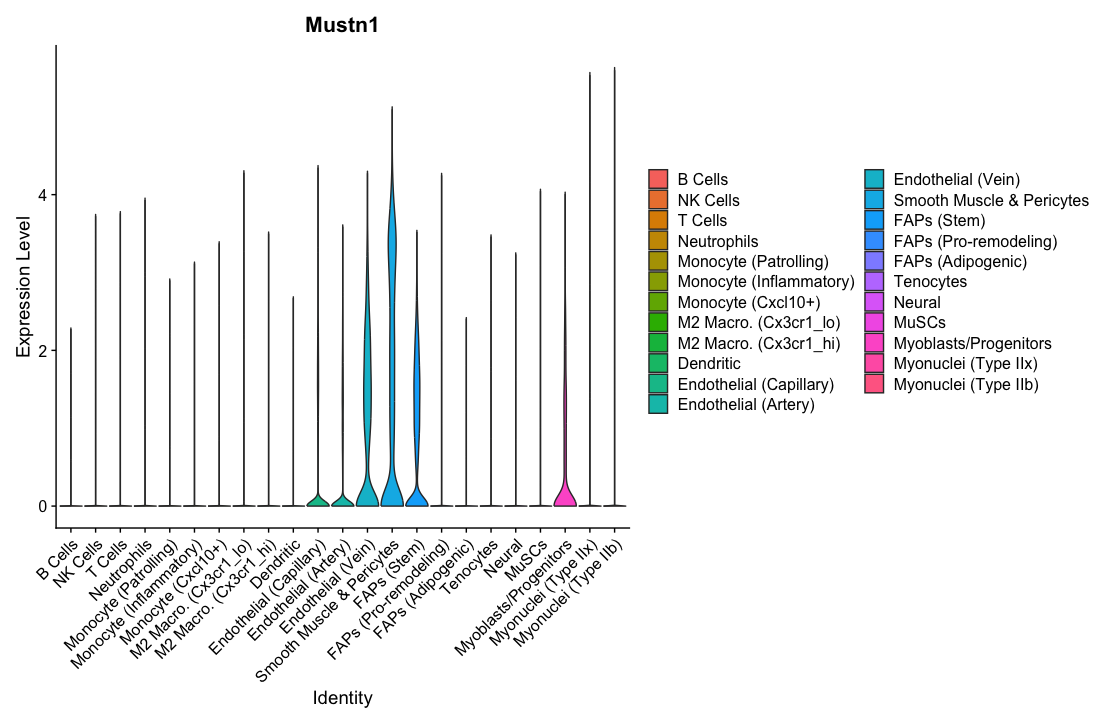

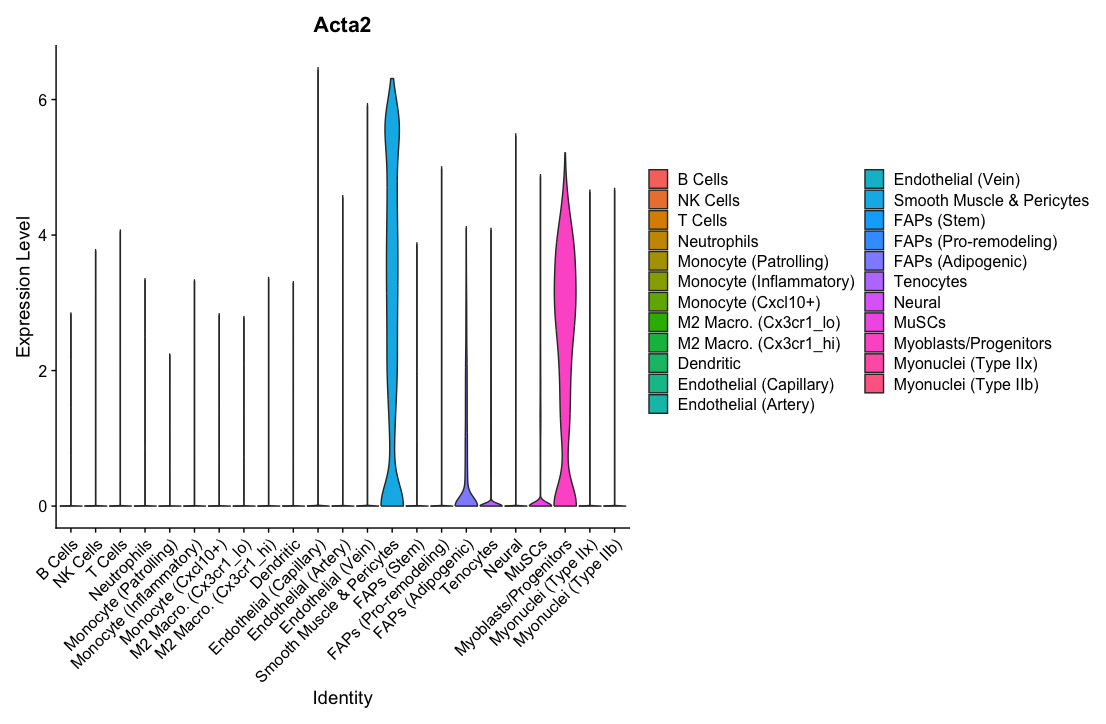


**Integrated dataset of Prx1-lineage cells from muscle adjacent to: 1) unfractured long bone d0 (sc3) 2) d3 (sc4), d5 (sc5) post-fracture 3) d3 (sc9), d5 (sc10) post-polytrauma^50^**

First, load the necessary Rstudio packages.

library(Seurat)
library(tidyverse)
library(Matrix)
library(data.table)

Then, create an integrated dataset from each timepoint. Plot pre-quality control metrics.

#read barcodes, features genes and matrix (ensure that the barcode/matrix/features file names are "barcode.tsv.gv", "matrix.tsv.gv" and "features.tsv.gv"
sc3 <- Read10X("/Users/chrisjanton/Downloads/GSE164573_RAW/sc3")
sc4 <- Read10X("/Users/chrisjanton/Downloads/GSE164573_RAW/sc4")
sc5 <- Read10X("/Users/chrisjanton/Downloads/GSE164573_RAW/sc5")
sc9 <- Read10X("/Users/chrisjanton/Downloads/GSE164573_RAW/sc9")
sc10 <- Read10X("/Users/chrisjanton/Downloads/GSE164573_RAW/sc10")

#create Seurat object for each timepoint
sc3 <- CreateSeuratObject(counts = sc3, project = "sc3", min.cells = 3, min.features = 200)
sc4 <- CreateSeuratObject(counts = sc4, project = "sc4", min.cells = 3, min.features = 200)
sc5 <- CreateSeuratObject(counts = sc5, project = "sc5", min.cells = 3, min.features = 200)
sc9 <- CreateSeuratObject(counts = sc9, project = "sc9", min.cells = 3, min.features = 200)
sc10 <- CreateSeuratObject(counts = sc10, project = "sc10", min.cells = 3, min.features = 200)

#merge all seurat objects into one meta-object
scBM.big <- merge(sc3, y = c(sc4, sc5, sc9, sc10), add.cell.ids = c("d0PF", "d3PF", "d5PF", "3dPT","5dPT"), project = "scBMPFaPT")

#load mitochondrial DNA percent into Seurat object
scBM.big <- PercentageFeatureSet(scBM.big, pattern = "^MT-", col.name = "percent.mt")

#view meta-Seurat object
scBM.big

## An object of class Seurat
## 19632 features across 34291 samples within 1 assay
## Active assay: RNA (19632 features, 0 variable features)

head(colnames(scBM.big))

## [1] "d0PF_AAACCTGAGTGAAGAG-1" "d0PF_AAACCTGAGTGTACGG-1"
## [3] "d0PF_AAACCTGCACATCCGG-1" "d0PF_AAACCTGCATACTACG-1"
## [5] "d0PF_AAACCTGGTCACCCAG-1" "d0PF_AAACCTGGTCTAGCGC-1"

tail(colnames(scBM.big))

## [1] "5dPT_TTTGTTGTCATTCTTG-1" "5dPT_TTTGTTGTCCATAGGT-1"
## [3] "5dPT_TTTGTTGTCGAGTCCG-1" "5dPT_TTTGTTGTCGGCAGTC-1"
## [5] "5dPT_TTTGTTGTCGGCCCAA-1" "5dPT_TTTGTTGTCTCTATAC-1"

#pre-QC filter data
VlnPlot(scBM.big, features = c("nFeature_RNA", "nCount_RNA", "percent.mt"), ncol = 3)


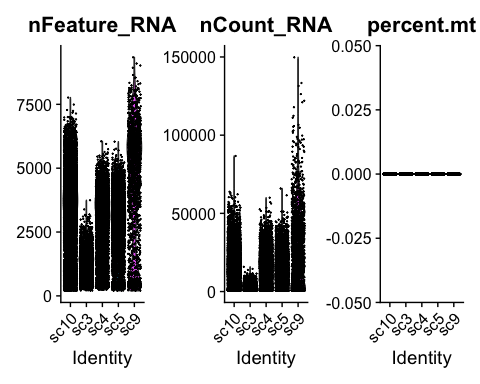


Next, quality control (QC) metrics were run as described in methods section and post-QC results are plotted.

scBM.big <- subset(scBM.big, subset = nFeature_RNA > 350 & nFeature_RNA <8000 & nCount_RNA < 20000)
VlnPlot(scBM.big, features = c("nFeature_RNA", "nCount_RNA", "percent.mt"), ncol = 3)


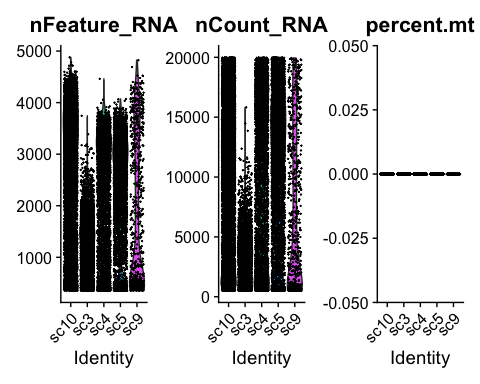


The data is then normalized logarithmically and the 2000 highest variable genes are identified.

#Data normalization
scBM.big <- NormalizeData(scBM.big, normalization.method = "LogNormalize", scale.factor = 10000)

#identify highly variable features
scBM.big <- FindVariableFeatures(scBM.big, selection.method = "vst", nfeatures = 2000)

Data scaling is applied and Principle Component Analyses (PCA) is performed. An elbow plot is used to determine the dimensionality of the data.

#data scaling
scBM.big <- ScaleData(scBM.big)
all.genes <- rownames(scBM.big)
scBM.big <- ScaleData(scBM.big, features = all.genes)

#Linear Dimensional reduction
#default setting: scTendonObject1 <- runPCA(scTendonObject1, npcs = 50, features = variablefeatures(object = scTendonObject1), ndims.print = 1:5, nfeatures.print = 30)
scBM.big <- RunPCA(scBM.big)
ElbowPlot(scBM.big, ndims = 50, reduction = "pca")


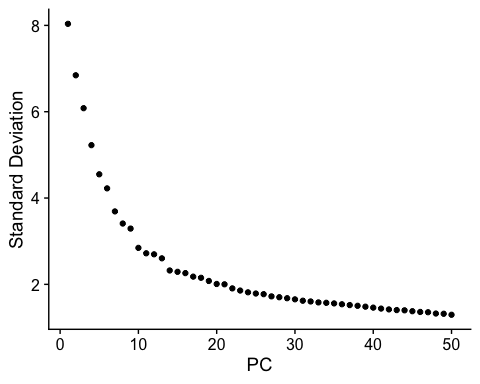


Cells are then clustered using PCA dimentionality of 10, as determined by hinge-point of elbow plot, in combination with Louvian algorithims. Uniform Manifold Approximation and Projection (UMAP) was used to visualize clustering in low-dimensional space. Clusters are labeled using published marker genes (see expression plots).

#clustering
scBM.big <- FindNeighbors(scBM.big, dims = 1:10)
scBM.big <- FindClusters(scBM.big, resolution = 0.1)

## Modularity Optimizer version 1.3.0 by Ludo Waltman and Nees Jan van Eck
##
## Number of nodes: 24420
## Number of edges: 776028
##
## Running Louvain algorithm...
## Maximum modularity in 10 random starts: 0.9666
## Number of communities: 7
## Elapsed time: 5 seconds

#non linear dimensional reduction UMAP/tSNE
scBM.big <- RunUMAP(scBM.big, dims = 1:10)
DimPlot(scBM.big, reduction = "umap", label = TRUE, repel = TRUE)


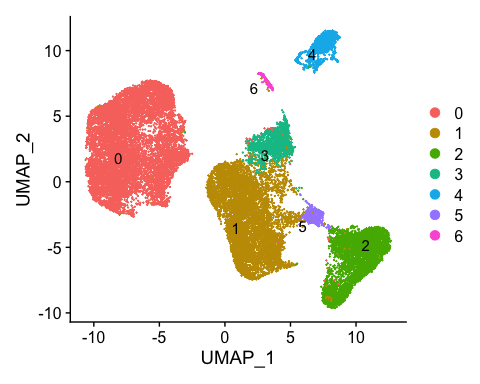


current.cluster.ids = c(0, 1, 2, 3, 4, 5, 6)
new.cluster.ids = c("FAP1", "FAP2", "FAP3", "NA", "Pericytes",
 "tendon ", "NA")
names(x = new.cluster.ids) <- levels(x = scBM.big)
scBM.big <- RenameIdents(object = scBM.big, new.cluster.ids)
DimPlot(scBM.big, reduction = "umap", label = TRUE, repel = TRUE, pt.size = 1) + NoLegend()


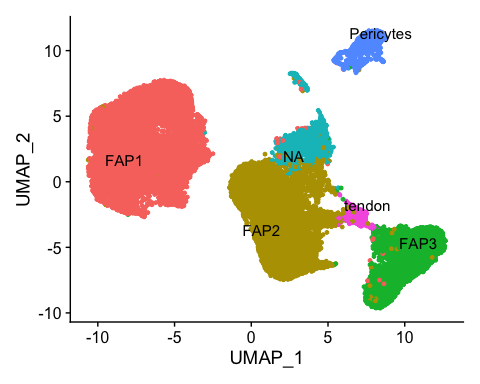


‘Mylk’,‘Des’,‘Cspg4’,‘Kera’,‘Scx’,‘Tnmd’,‘Prrx1’,‘Cxcl12’,‘Pdgfra’,‘Ly6a’,‘Cd34’,‘Mustn1’ coexpression plotted via dot plot.

DotPlot(scBM.big, features = c('Mylk','Des','Cspg4','Kera','Scx','Tnmd','Prrx1','Cxcl12','Pdgfra','Ly6a','Cd34','Mustn1'))


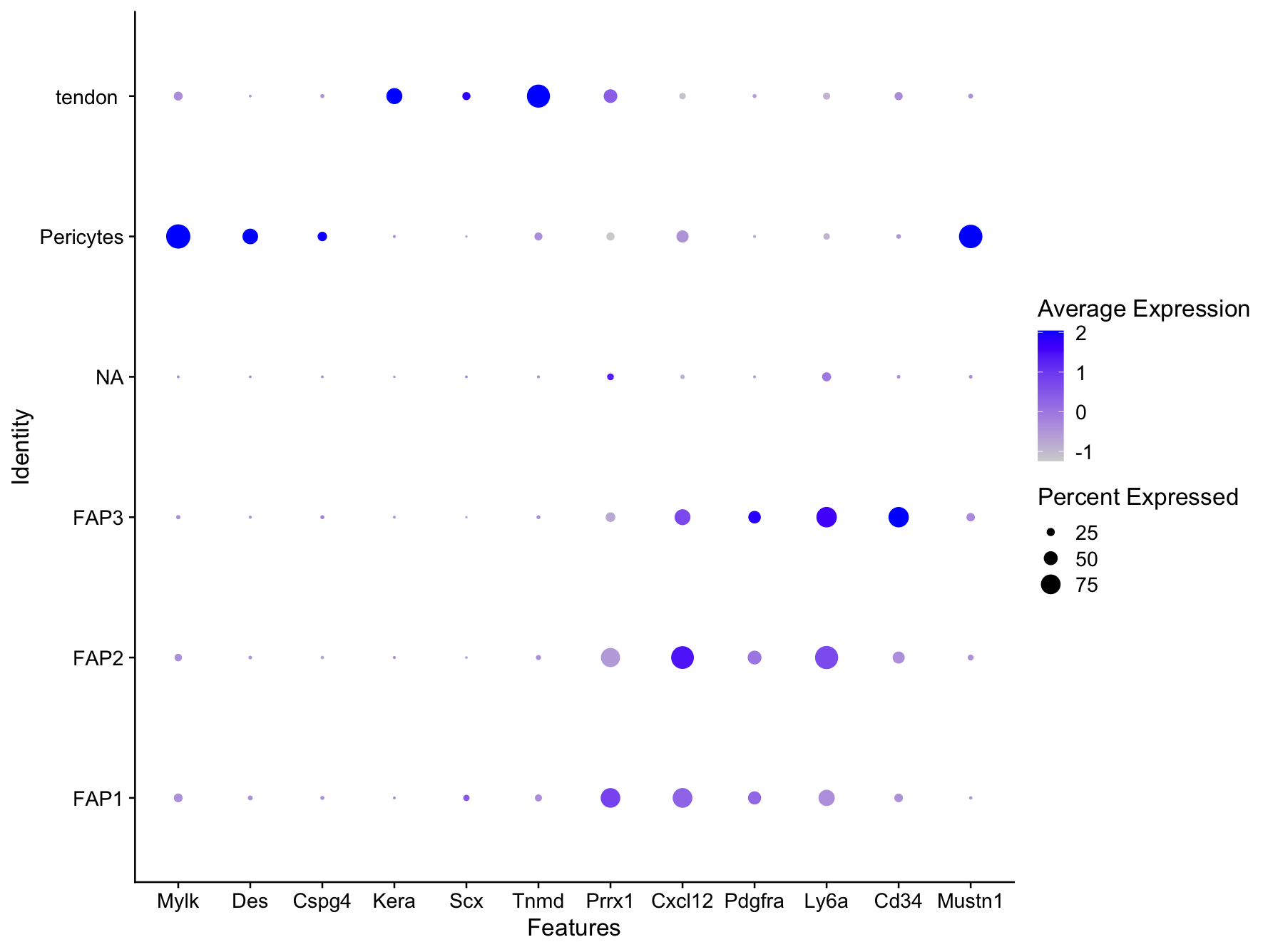


**sox9-lineage cells of p21 - p23, p28, p35 and p56 pulse-chased mouse hindlimbs^46^**

load proper Python packages in Jupyter Lab

import pegasus as pg
import scanpy as sc
import scvelo as scv
scv.settings.set_figure_params('scvelo')

Load annotated data object.

path = '/path/to/file/filtered2_data.h5ad'
NonImmune = pg.read_input(path)
NonImmune = NonImmune.to_anndata()

Plot gene expression on umap and violin plots.

sc.pl.umap(NonImmune, color = ['TDTOMATO','Sox9','Mustn1','Mylk','Des','Acta2','Mcam','Scx','Tnmd','Cxcl12','Pdgfra','Ly6a','Cd34','Prg4'], size=50, color_map='YlGnBu', legend_fontsize=15, vmax = '10')
sc.pl.stacked_violin(NonImmune, ['TDTOMATO','Sox9','Mustn1','Mylk','Des','Acta2','Mcam','Scx','Tnmd','Cxcl12','Pdgfra','Ly6a','Cd34','Prg4'], groupby='new_clusters', size = 0, row_palette='darkblue', swap_axes=True)
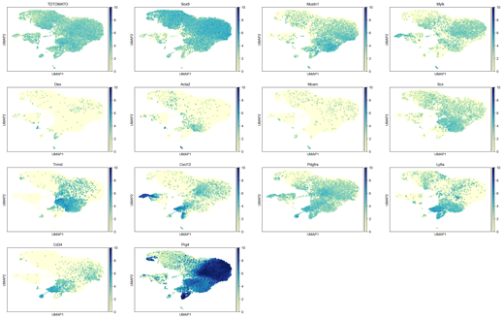

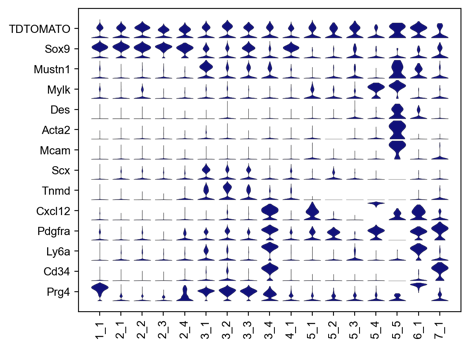

Supplement: Supplementary_Methods_ziaf193 [file supplementary_methods_ziaf193.docx]
